
JOURNAL INFORMATION
==============================
NLM Title Abbreviation: J Can Health Libr Assoc
Journal ID: J Can Health Libr Assoc
NLM Journal ID: 101229936
Journal ID: JCHLA

The Journal of the Canadian Health Libraries Association

EISSN: 1708-6892
Publisher: Canadian Health Libraries Association / Association des bibliotèques de la santé du Canada

ARTICLE INFORMATION
==============================
PMCID: PMC10403111
DOI: 10.29173/jchla29722
Article ID: JCHLA-44-070
Article version: 1
Subjects: Abstracts

CHLA 2023 CONFERENCE POSTERS / ABSC CONGRÈS 2023 AFFICHES

Electronic publication date: 2023 Aug 1
Publication date: 2023 Aug
Volume: 44
Issue: 2
First page: 70
Last page: 75
License: This article is distributed under a Creative Commons Attribution License: https://creativecommons.org/licenses/by/4.0/
License URL: https://creativecommons.org/licenses/by/4.0/

==============================

PP = Poster Presentation

## PP1. Required, not suggested: Creating a rigorous protocol template for improved uptake and efficacy

Sabine Calleja, Carolyn Zeigler
Unity Health

**Introduction:** With knowledge synthesis (KS) projects becoming increasingly popular research projects, it is not uncommon that many are poorly conceived. Protocols are an integral planning component to the KS process, so why are so many below par and not well-thought out? As information specialists collaborating on almost 40 KS projects a year, we frequently encounter research teams that believe the review starts with the search and simultaneously our roles have expanded to providing extensive feedback on protocols. This poster outlines the outcomes and uptake of an improved, user-focused KS protocol template following its pilot-testing process, workshop demonstration, and its launch on a web-based platform. **Description:** A rigorous revision of a hospital library team's KS protocol template was completed in January 2023. To ensure the success and uptake of the new protocol template, a pilot-testing process among key researchers in the organization will be undertaken and a workshop demonstration and explanation will be conducted. It is anticipated that the revised KS protocol will create a more sustainable KS workflows for the library team. **Outcomes:** Feedback from clients and statistics on the uptake of the new KS protocol template will be shared. The presenters will share how the improved protocol template has impacted time spent on revising protocols. Attendees will learn how to improve their own protocol templates to ensure more sustainable KS collaborations. **Discussion:** This poster will discuss how to evaluate and successfully implement changes to a protocol template so that KS collaborations are more efficient and produce higher-quality work.

## PP2. A Hot Topic: Burnout in Health Sciences Literature, 2012-2022

Madelaine Hare, Courtney Svab
Dalhousie University

**Introduction:** The health sciences have devoted increasing attention to burnout due to the distinctive physical and psychological demands of medical professions, high performance requirements, and insufficient nature of governmental support to alleviate them, which manifest in negative consequences for working professionals. Studies using bibliometric methods that overview burnout in the Health Sciences have traditionally been undertaken at the disciplinary level. This field-level study seeks to answer the following research questions: RQ 1. 1. What clusters of burnout research are identified in health sciences literature? RQ 2. 1. How many research papers on burnout are being published in Health Sciences literature overall and in the identified clusters? 2.2. How has the output of literature on burnout changed over time? RQ 3. How has the literature on burnout changed since the beginning of the COVID-19 pandemic? **Methods:** Journal articles on burnout from PubMed Central, Ovid Medline, Embase, CINAHL, PsycInfo, and Cochrane Library were retrieved for the period 2012- 2022. Citation relationships were used to cluster articles based on top journals and keywords to create a network of literature. **Results:** We identify seven clusters in the health sciences: Point of Care, Education and Training, Psychological Wellbeing, Public Health and Research, Sports Health, Bioresources, Science, &

Technology, and Oncology. **Discussion:** The Point of Care cluster represents 33.57% of the literature, the largest proportion amongst the clusters. We find a significant increase on burnout literature resulting from the COVID-19 pandemic. We provide suggestions for future approaches to burnout research, including ways librarians can support researchers studying burnout, and how to use findings from this analysis to situate new studies.

## PP3. The Journal Selector Tool

Margaret Hoogland
University of Toledo

**Introduction:** Faculty, learners, and staff request assistance from health science librarians and informationists for locating journals. Sometimes they have specific journals or submission categories in mind. At other times, they focus on the journal impact factor, open access, and publication fees. Regardless of the request, the informationist or librarian does a search and provides suggestions. **Description:** The journal selector tool consists of the journal title, journal abbreviation, primary submission categories (review, case study, case report, research, etc.), publication costs (e.g, no cost, cost under $1000, cost between $1000-3000, cost above $3000), Journal Impact Factor, and Journal Citation Indicator Score. Due to proprietary content, the author created a dedicated and password protected LibGuide for this tool. With this tool, the learner, faculty, or staff member can begin searching for a journal by themselves, if the librarian or informationist is unavailable. **Outcomes:** This tool provides Faculty, staff, and learners with a faster and more reliable way to compare and contrast publication options. Further, the author can encourage prospective submitters to consider more than just the Impact Factor, when selecting a journal. While the author uses REDCap and Libguides, other librarians and informationists could create a similar tool using Microsoft Access, free AirTable, or even Excel. While updating the tool takes time, the author feels use and support for the end product justifies the time required for maintaining the tool. **Discussion:** Arguments could be made that the Journal Selector Tool minimizes the role of the informationist or librarian. The counter argument is that marketing such a tool could connect the librarian and informationists to new members of the community, because this tool meets an existing and future need.

## PP4. Partner in Innovation Journey

Alla Iansavitchene, Maureen Doherty Nielsen
London Health Sciences Centre

**Introduction:** Innovation and change are a part of everyday life. This is especially true for the hospital librarian supporting the unique needs of healthcare providers in a fast-moving environment where technological innovations and new clinical practices get ongoing adoption. However, a lack of support and training in leading innovation in healthcare settings for librarians can inhibit creativity and new idea generation. **Description:** This paper aims to discuss the journey of our Library to bring innovation into practice through the participation of one Clinical Librarian in a brand-new six-month-long comprehensive training program, the "Discoveries in Leading Innovation" offered at London Health Sciences Centre (LHSC). **Results/Outcomes:** Details, the application process, benefits of entering the program, and insights gained from the participation in a cohort of hospital staff, who took part in this in-house program at LHSC, are discussed. **Discussion:** Libraries and library staff need to grow and innovate and start taking steps to practice creativity. The in-house program can help to prepare library staff to assume leadership in innovation roles with confidence and competence, making them more likely to succeed in achieving innovative outcomes. It is an empowering thing to do.

## PP5. Developing a machine learning tool to optimize literature surveillance in PubMed

Tamara Navarro-Ruan[1], Patricia Kavanagh, Peter LaVita[2], Rick Parrish[1], Lori Linkins[1], Alfonso Iorio[1]
[1]McMaster University; [2]EBSCO Health

**Introduction:** Despite recent advances in the use of artificial intelligence (AI) to automate literature surveillance, initiatives incorporating AI tools into library processes remain quite uncommon. The Health Information Research Unit (HiRU) has developed a machine learning (ML) Classifier tool to increase efficiency in capturing high-quality clinical evidence. **Description:** The HiRU PLUS database is comprised of clinical research articles surveyed daily from 120 PubMed clinical journals and checked for methodological rigor by expert Research Associates. Over 150,000 records from the PLUS database from 2012 to 2020 have been used to derive and validate an ML algorithm to predict high-quality clinical content. Rare topics, such as those captured by the DynaMed Systematic Literature Surveillance (SLS) process, are challenging to keep current due to the large volume of articles that must be reviewed to find the few high-quality ones of interest. Working from content-based PubMed search strategies for 647 clinical topics within the SLS database, the HiRU ML tool was applied to provide probability rankings on the likelihood of articles passing the criteria for high-quality. **Outcomes:** The HiRU ML tool ranked 9,163 out of 231,558 retrieved articles (3%) as high probability for being of high-quality. Out of the 9,163 articles, 858 have been selected by clinical experts to update 320 topics, eliminating the need for manual review of 222,395 articles. **Discussion:** The probability-ranking ML tool can be used to optimize efficiency in the literature surveillance process for rare topics. The probably ranking feature of the ML Classifier can be used by librarians and health information researchers to identify higher-quality content in a timely and efficient manner.

## PP6. 'Available upon reasonable request': Search strategy sharing statements and practices in published systematic reviews

Christine Neilson[1], Zahra Premji[2]
[1]University of Manitoba; [2]University of Victoria

**Introduction:** There has been an increased emphasis on research transparency and data sharing in recent years. The Preferred Reporting Items for Systematic Reviews and Meta-analysis (PRISMA) guideline was created in 2009, and updated in 2021, to improve transparency of published systematic reviews (SRs). The PRISMA checklist clearly states that complete search strategies should be reported but authors may not adhere to the guideline. A 2017 scoping review on PRISMA compliance showed that the pooled adherence to item #8 (inclusion of search strategy) of the PRISMA checklist was ~62% (Page & Moher, 2017). Some review authors opt to include a statement that the search strategy is available on request, rather than publish them with the review manuscript. This study examined published SRs containing search strategy availability statements and their subsequent sharing practices. **Methods:** We conducted fulltext searches using Google Scholar, Lens.org, Academic Search Complete, and EBSCO Medline to identify English-language SRs containing search availability statements that were published in the past five years. Results were deduplicated and screened using Covidence software. **Results:** 155 SRs were included for data extraction. While some authors readily shared their search strategies, others did not. Reproducibility of the shared strategies varied. Failure to share search strategies reduces transparency and may bring the quality of the overall work into question. Page, M. J., & Moher, D. (2017). Evaluations of the uptake and impact of the Preferred Reporting Items for Systematic reviews and Meta-Analyses (PRISMA) Statement and extensions: a scoping review. Systematic reviews, 6(1), 263. doi: 10.1186/s13643-017-0663-8

## PP7. Scaffolding IL learning and EBP exploration in a semester-long journal club: Impact on nursing student self-efficacy

Jody Nelson, Hanneke Croxen, Lisa McKendrick-Calder, Lam Ha, Wanhua Su
MacEwan University

**Background:** Information Literacy (IL) skill development supports academic success for nursing students and is foundational to Evidence Based Practice (EBP) for nursing graduates. Bachelor of Science in Nursing (BScN) Students at our mid-sized university are expected to develop competencies in locating, evaluating, interpreting and applying relevant literature, receiving targeted IL instruction from the embedded subject librarian beginning in their first-year theory course. **Description:** In 2020 the theory instructor and subject librarian developed and initiated a new model for greater IL integration, scaffolding IL learning with semester-long, discovery-based, group journal clubs. The redesign sought to address three known issues for students: 1) limitations of IL skill development through the one-shot library instruction model 2) difficulties in reading and applying published research, and 3) challenges of developing an EBP mindset. A scholarship of teaching and learning study was undertaken to identify the impact of our novel journal club approach on student IL self-efficacy as measured through the validated Information Literacy Self-Efficacy Scale (ILSES) developed by Kurbanoglu et al. (2006). **Methods:** An assessment of learning was carried out in Fall 2022 using a pre- / post- study design to measure the impact of the semester-long journal club on students' self-efficacy in relation to core IL competencies. The ILSES was administered to students at the start of the semester, prior to the first journal club class, and at the end of the semester, following the 6th and final journal club class.

## PP8. Usability and potential impact of a literacy-oriented intervention for patients with complex care needs: A case study

Pierre Pluye[1], Virginie Paquet[2], Vera Granikov[1], Francesca Frati[1], Fabio Balli[3], Carrie Dai[1], Reem Ell Sherif[1], Quan Nha Hong[2], Rolan Grad[1]
[1]McGill University; [2]Universite de Montreal; [3]Concordia University

**Introduction:** Community-dwelling patients with complex care needs (hereafter 'patients') seek, evaluate and use information to choose optimal care. However, patients with low eHealth literacy often have difficulty finding trustworthy information easy to read, listen to, or watch. Improving their eHealth literacy can: increase self-care and participation in healthcare; decrease health problems, use and cost of services; and reduce inequalities. **Objectives:** Describe the usability and potential impact of a web-based ehealth literacy-oriented intervention. **Methods:** An exploratory case report was performed. Intervention: To help patients find, appraise and use online health information (three principles of ehealth literacy), an innovative website (Online Health Information Aid: OHIA) and an educational video with a game were created. Five low SES patients received the intervention (OHIA-website-video-game). Pre-intervention (J1) and post-intervention (J30) data were collected. Quantitative and qualitative data were analysed using descriptive statistics and thematic analysis, respectively. Quantitative results and qualitative findings were compared (joint-display). **Results:** Participants were three women and two men aged 46 to 71 years (mean 62) with low level of education and revenue, two to 11 chronic health problems (mean 5), and two to 20 drugs (mean 10). They found the website usable (e.g., 'good tool'). Regarding the video, the usability score was high (67% to 96%; mean 79%) and comments were positive (e.g., 'good and helpful'). However, the usability of the game was lower (40% to 78%; mean 60%) and comments were negative (e.g., 'complex and not readable'). For three participants, the level of ehealth literacy (n=2) and/or the knowledge for appraising online health information (n=2) increased post-intervention. However, they did not perceive any intervention's impact. **Conclusion:** Results suggest that the OHIA-website-video constitutes a promising intervention.

## PP9. COVID-19 journal articles indexing in web-scale discovery services: An event history analysis

Michelle Swab
Memorial University

**Introduction:** Although health librarians tend to use subject databases for searching, web-scale discovery services (WSDS) are increasingly integrated with library services platforms to facilitate full-text article access and interlibrary loan services. Therefore, the speed of journal article indexing remains an important feature of WSDS. This study analyzes the length of time to indexing of COVID-19 journal articles during two time periods in 2020. **Methods:** COVID-19 related citations were downloaded from PubMed on a daily basis during two periods: Feb. 15th to 29th 2020 and Nov. 14th to 28th 2020. Citations were searched by both title and digital object identifier in each WSDS until indexed or until 15 days had been reached. To mitigate issues with full-text availability limiting retrieval, each search was expanded beyond the local library. The following WSDS were included in the study: Ex Libris Primo, Ex Libris Summon, EBSCO Discovery Services, and OCLC Worldcat Discovery. Google Scholar was also included as a comparator. **Results:** Average time to indexing in the February 2020 period ranged from 0.9 days (Worldcat Discovery) to 5.5 days (Primo) for articles that were indexed within the 15 day time period. Average time to indexing in the November 2020 period ranged from 0.7 days (Worldcat Discovery) to 4 days (Primo) for articles that were indexed within the 15 day time period. **Discussion:** While WSDS are widely used within academic libraries, the speed and frequency of journal article updates in WSDS indexes remains somewhat opaque. This study provides insights into the lag between article publication and time to indexing in major WSDS.

## PP10. Tiny? Make it mighty! Maximizing a limited-budget upgrade of a pint-sized hospital library using UX methods

**Introduction:** The University of Ottawa Heart Institute's Berkman Library space is outdated. Budget constraints and tiny square footage leave little room for error or experimentation. A needs assessment was conducted from 2022-2023 to inform strategic decisions on updating and reorganizing furnishings to better support users and their varied needs. **Description:** Data was collected via an electronic survey of hospital staff, in-situ interviews, user observations, and a physical survey of alternative communal spaces in the building. Resulting numeric, narrative, and pictorial data were compiled and examined for common themes. Low fidelity mock-ups of furnishings and arrangements were prototyped and presented to users for feedback. **Outcomes:** Quiet was one of the most valued attributes of the space. The physical survey of communal spaces revealed that research staff had little access to comfortable space, clinical staff had little access to quiet space, and neither group had access to desktop computers out of public view. Survey and in-situ interview responses consistently cited soft, comfortable furnishings as desirable additions to the workspaces currently offered. Observed behaviours support the continued need for desks with a deep surface area to accommodate multiple devices used in tandem. Flexible use of computer hardware, more accessible power outlets, and adjustable lighting were identified as additional gaps. **Discussion:** Results contextualize the library's space, identify qualities that address institutional gaps, and provide insight into the motivators, needs, and behaviours of hospital staff. Centering user behaviours and preferences in the project's methodology has provided data that supports both near-term decision-making for physical upgrades and long-term library policy.

## PP11. The role of paywalls in the online information-seeking behaviour of Canadian midwives: Preliminary results

Richmond Yeboah, Joan Bartlett
McGill University

**Introduction:** Past research has found paywalls to be a major barrier to Canadian midwives' online information access for practice. However, studies on how paywalls affect information-seeking behaviour are limited and unavailable in the case of Canadian midwives. Moreover, there are inconsistent findings on this subject: some studies revealed a decline in use of sources because of paywalls, while others showed an increase in perceived value and source selection when paywalls were introduced. As part of a larger study to understand how paywalls affect the information-seeking behaviour of Canadian midwives, we present preliminary findings of research into the perceived value of premium online health information sources compared to free ones. **Methods:** In this phase, a web-based questionnaire was designed based on established and tested scales assessing perceived value of information resources. The survey will be administered through the Canadian Association of Midwives' email list. Questions include the perceptions of quality, reliability, trustworthiness, ease of use and intention to use premium and free online information sources. Analysis will include descriptive statistics, and ANOVA mean comparisons. **Results:** This study is in progress. Preliminary results will be presented.

JOURNAL INFORMATION
==============================
NLM Title Abbreviation: J Can Health Libr Assoc
Journal ID: J Can Health Libr Assoc
NLM Journal ID: 101229936
Journal ID: JCHLA

The Journal of the Canadian Health Libraries Association

EISSN: 1708-6892
Publisher: Canadian Health Libraries Association / Association des bibliotèques de la santé du Canada

ARTICLE INFORMATION
==============================
Subjects: PP = Poster Presentation

PP1. Required, not suggested: Creating a rigorous protocol template for improved uptake and efficacy

Calleja Sabine
Zeigler Carolyn
Unity Health

JOURNAL INFORMATION
==============================
NLM Title Abbreviation: J Can Health Libr Assoc
Journal ID: J Can Health Libr Assoc
NLM Journal ID: 101229936
Journal ID: JCHLA

The Journal of the Canadian Health Libraries Association

EISSN: 1708-6892
Publisher: Canadian Health Libraries Association / Association des bibliotèques de la santé du Canada

ARTICLE INFORMATION
==============================
Subjects: PP = Poster Presentation

PP2. A Hot Topic: Burnout in Health Sciences Literature, 2012-2022

Hare Madelaine
Svab Courtney
Dalhousie University

JOURNAL INFORMATION
==============================
NLM Title Abbreviation: J Can Health Libr Assoc
Journal ID: J Can Health Libr Assoc
NLM Journal ID: 101229936
Journal ID: JCHLA

The Journal of the Canadian Health Libraries Association

EISSN: 1708-6892
Publisher: Canadian Health Libraries Association / Association des bibliotèques de la santé du Canada

ARTICLE INFORMATION
==============================
Subjects: PP = Poster Presentation

PP3. The Journal Selector Tool

Hoogland Margaret
University of Toledo

JOURNAL INFORMATION
==============================
NLM Title Abbreviation: J Can Health Libr Assoc
Journal ID: J Can Health Libr Assoc
NLM Journal ID: 101229936
Journal ID: JCHLA

The Journal of the Canadian Health Libraries Association

EISSN: 1708-6892
Publisher: Canadian Health Libraries Association / Association des bibliotèques de la santé du Canada

ARTICLE INFORMATION
==============================
Subjects: PP = Poster Presentation

PP4. Partner in Innovation Journey

Iansavitchene Alla
Nielsen Maureen Doherty
London Health Sciences Centre

JOURNAL INFORMATION
==============================
NLM Title Abbreviation: J Can Health Libr Assoc
Journal ID: J Can Health Libr Assoc
NLM Journal ID: 101229936
Journal ID: JCHLA

The Journal of the Canadian Health Libraries Association

EISSN: 1708-6892
Publisher: Canadian Health Libraries Association / Association des bibliotèques de la santé du Canada

ARTICLE INFORMATION
==============================
Subjects: PP = Poster Presentation

PP5. Developing a machine learning tool to optimize literature surveillance in PubMed

Navarro-Ruan Tamara 1
Kavanagh Patricia
LaVita Peter 2
Parrish Rick 1
Linkins Lori 1
Iorio Alfonso 1
1 McMaster University
2 EBSCO Health

JOURNAL INFORMATION
==============================
NLM Title Abbreviation: J Can Health Libr Assoc
Journal ID: J Can Health Libr Assoc
NLM Journal ID: 101229936
Journal ID: JCHLA

The Journal of the Canadian Health Libraries Association

EISSN: 1708-6892
Publisher: Canadian Health Libraries Association / Association des bibliotèques de la santé du Canada

ARTICLE INFORMATION
==============================
Subjects: PP = Poster Presentation

PP6. 'Available upon reasonable request': Search strategy sharing statements and practices in published systematic reviews

Neilson Christine 1
Premji Zahra 2
1 University of Manitoba
2 University of Victoria

JOURNAL INFORMATION
==============================
NLM Title Abbreviation: J Can Health Libr Assoc
Journal ID: J Can Health Libr Assoc
NLM Journal ID: 101229936
Journal ID: JCHLA

The Journal of the Canadian Health Libraries Association

EISSN: 1708-6892
Publisher: Canadian Health Libraries Association / Association des bibliotèques de la santé du Canada

ARTICLE INFORMATION
==============================
Subjects: PP = Poster Presentation

PP7. Scaffolding IL learning and EBP exploration in a semester-long journal club: Impact on nursing student self-efficacy

Nelson Jody
Croxen Hanneke
McKendrick-Calder Lisa
Ha Lam
Wanhua Su MacEwan University

JOURNAL INFORMATION
==============================
NLM Title Abbreviation: J Can Health Libr Assoc
Journal ID: J Can Health Libr Assoc
NLM Journal ID: 101229936
Journal ID: JCHLA

The Journal of the Canadian Health Libraries Association

EISSN: 1708-6892
Publisher: Canadian Health Libraries Association / Association des bibliotèques de la santé du Canada

ARTICLE INFORMATION
==============================
Subjects: PP = Poster Presentation

PP8. Usability and potential impact of a literacy-oriented intervention for patients with complex care needs: A case study

Pluye Pierre 1
Paquet Virginie 2
Granikov Vera 1
Frati Francesca 1
Balli Fabio 3
Dai Carrie 1
Sherif Reem Ell 1
Hong Quan Nha 2
Grad Rolan 1
1 McGill University
2 Universite de Montreal
3 Concordia University

JOURNAL INFORMATION
==============================
NLM Title Abbreviation: J Can Health Libr Assoc
Journal ID: J Can Health Libr Assoc
NLM Journal ID: 101229936
Journal ID: JCHLA

The Journal of the Canadian Health Libraries Association

EISSN: 1708-6892
Publisher: Canadian Health Libraries Association / Association des bibliotèques de la santé du Canada

ARTICLE INFORMATION
==============================
Subjects: PP = Poster Presentation

PP9. COVID-19 journal articles indexing in web-scale discovery services: An event history analysis

Swab Michelle
Memorial University

JOURNAL INFORMATION
==============================
NLM Title Abbreviation: J Can Health Libr Assoc
Journal ID: J Can Health Libr Assoc
NLM Journal ID: 101229936
Journal ID: JCHLA

The Journal of the Canadian Health Libraries Association

EISSN: 1708-6892
Publisher: Canadian Health Libraries Association / Association des bibliotèques de la santé du Canada

ARTICLE INFORMATION
==============================
Subjects: PP = Poster Presentation

PP10. Tiny? Make it mighty! Maximizing a limited-budget upgrade of a pint-sized hospital library using UX methods

Visintini Sarah 1
McEwan Jessica 2
1 University of Ottawa Heart Institute
2 University of Ottawa

JOURNAL INFORMATION
==============================
NLM Title Abbreviation: J Can Health Libr Assoc
Journal ID: J Can Health Libr Assoc
NLM Journal ID: 101229936
Journal ID: JCHLA

The Journal of the Canadian Health Libraries Association

EISSN: 1708-6892
Publisher: Canadian Health Libraries Association / Association des bibliotèques de la santé du Canada

ARTICLE INFORMATION
==============================
Subjects: PP = Poster Presentation

PP11. The role of paywalls in the online information-seeking behaviour of Canadian midwives: Preliminary results

Yeboah Richmond
Bartlett Joan
McGill University

